# Functional Expression of TWEAK and the Receptor Fn14 in Human Malignant Ovarian Tumors: Possible Implication for Ovarian Tumor Intervention

**DOI:** 10.1371/journal.pone.0057436

**Published:** 2013-03-04

**Authors:** Liying Gu, Lan Dai, Cong Cao, Jing Zhu, Chuanwei Ding, Hai-bo Xu, Lihua Qiu, Wen Di

**Affiliations:** 1 Department of Gynecology and Obstetrics, Renji Hospital, Shanghai JiaoTong University School of Medicine, Shanghai, P.R. China; 2 Shanghai Key Laboratory of Gynecologic Oncology, Shanghai, P.R. China; 3 The 2^nd^ Affiliated Hospital of Soochow University, Suzhou, P.R. China; 4 Gynecologic Oncology, Affiliated Tumor Hospital of Nantong University, Nantong, P.R. China; Children’s National Medical Center, Washington, United States of America

## Abstract

The aim of this current study was to investigate the expression of the tumor necrosis factor (TNF)-like weak inducer of apoptosis (TWEAK) and its receptor fibroblast growth factor-inducible 14 (Fn14) in human malignant ovarian tumors, and test TWEAK’s potential role on tumor progression in cell models *in-vitro*. Using immunohistochemistry (IHC), we found that TWEAK and its receptor Fn14 were expressed in human malignant ovarian tumors, but not in normal ovarian tissues or in borderline/benign epithelial ovarian tumors. High levels of TWEAK expression was detected in the majority of malignant tumors (36 out of 41, 87.80%). Similarly, 35 out of 41 (85.37%) malignant ovarian tumors were Fn14 positive. In these malignant ovarian tumors, however, TWEAK/Fn14 expression was not corrected with patients’ clinical subtype/stages or pathological features. *In vitro,* we demonstrated that TWEAK only inhibited ovarian cancer HO-8910PM cell proliferation in combination with tumor necrosis factor-α (TNF-α), whereas either TWEAK or TNF-α alone didn’t affect HO-8910PM cell growth. TWEAK promoted TNF-α production in cultured THP-1 macrophages. Meanwhile, conditioned media from TWEAK-activated macrophages inhibited cultured HO-8910PM cell proliferation and invasion. Further, TWEAK increased monocyte chemoattractant protein-1 (MCP-1) production in cultured HO-8910PM cells to possibly recruit macrophages. Our results suggest that TWEAK/Fn14, by activating macrophages, could be ovarian tumor suppressors. The unique expression of TWEAK/Fn14 in malignant tumors indicates that it might be detected as a malignant ovarian tumor marker.

## Introduction

The tumor necrosis factor (TNF)-like weak inducer of apoptosis (TWEAK) was first described as a member of the tumor necrosis factor (TNF) superfamily in 1997 [1]. TWEAK was initially identified as a weak inducer of apoptosis in transformed cell lines [2], [3]. It is now well-accepted that TWEAK is a multifunctional cytokine depending on conditions and cell types [4], soluble TWEAK stimulates murine astrocytes and human HepG2 cells proliferation [5], [6], murine RAW264.7 cell differentiation [7], and induces HSC3 cell death [8]. TWEAK could also stimulate angiogenesis and inflammatory cytokines production [4]. Lynch et al. reported that picomole concentrations of recombinant soluble TWEAK induces the proliferation of a variety of normal human endothelial cells, and reduced the requirements for serum and growth factors during the culture of aortic smooth muscle cells [9].

TWEAK expression is observed in multiple tumors including breast tumors, human hepatocellular carcinoma (HCC), colon carcinoma and glioblastoma multiforme, as well as in multiple tumor cell lines [6], [10], [11], [12], [13]. Fibroblast growth factor-inducible 14 (Fn14), the TWEAK receptor [14], is also expressed in multiple tumor tissues [15], [16]. Studies have suggested that TWEAK and Fn14 might be associated with tumorigenesis [4], [17]. Meanwhile, researchers have found that TWEAK may have anti-tumor effects [18], [19], [20], [21], [22]. The expression of TWEAK/Fn14 and their potential function in ovarian tumors are not fully defined.

In this study, we determined the expression of the TWEAK and its receptor Fn14 in human malignant ovarian tumors, and test TWEAK’s potential role on tumor progression in *in-vitro* cell models. Additionally, we aimed to understand how TWEAK affects innate immunity during tumorigenesis. The present study explored the effect of TWEAK on macrophages, and the subsequent effects of TWEAK and macrophage-derived tumor necrosis factor-α (TNF-α) on ovarian cancer cell proliferation and metastasis.

## Materials and Methods

### Patients and Ethics

Formalin-fixed paraffin-embedded ovarian tumors (including 41 malignant tumor tissues and 20 borderline or benign tumors) and normal ovarian tissues were selected from the archives at the department of Obstetrics & Gynecology at Renji Hospital, Shanghai JiaoTong University School of Medicine (Shanghai, China), from 2000 to 2007. The study was approved by the institutional review board of Renji Hospital, Shanghai JiaoTong University School of Medicine, and written informed consent was obtained from all patients. All clinical investigation was conducted according to the principles expressed in the Declaration of Helsinki.

### Immunohistochemistry

Immunohistochemistry (IHC) was performed using the horseradish peroxidase (HRP)-polymer anti-mouse IHC DAB (diaminobenzidine)-based kit (MaxVision, Fuzhou, China), according to the manufacturer protocol. Antigen retrieval was performed using borate buffer (pH = 8), followed by incubation in hydrogen peroxide and additional blocking steps. Anti-TWEAK and anti-Fn14 primary antibodies were purchased from Santa Cruz Biotechnology (Santa Cruz, CA) and used at 1∶50. The IHC was examined and imaged using an OLYMPUS BX51 microscope (Tokyo, Japan) at 1∶200.

### Cell Culture

The highly metastatic human ovarian cancer HO-8910PM cell line was obtained from the Cell Bank of the Chinese Academy of Sciences (Shanghai, China) [23], [24], [25]. Cells were cultured in Roswell Park Memorial Institute 1640 medium (RPMI 1640; Gibco, NY) supplemented with 10% fetal bovine serum (FBS, Gibco) and penicillin/streptomycin (1∶100, Sigma, St. Louis, MO), in a humidified atmosphere containing 5% CO_2_ at 37°C. Unless otherwise indicated, the cells were cultured to 70∼80% confluence, cells were then serum-starved overnight in serum-free RPMI1640 media prior to treatments. The human monocyte cell line THP-1 was obtained from the ATCC (Manassas, VA) and cultured in RPMI 1640 supplemented with 10% FBS and penicillin/streptomycin (1∶100), in a humidified atmosphere containing 5% CO_2_ at 37°C.The THP-1 cell was treated with 100 ng/ml PMA (Phorbol-12-myristate-13-acetate) for differentiation [26].

### Chemicals and Reagents

Human recombinant TWEAK was purchased from PeproTech (Rock Hill, NJ). BD Matrigel™ Basement Membrane Matrix (5 ml; LDEV-Free) was purchased from Becton Dickinson (Franklin Lakes, NJ). The Millicell Hanging Cell Culture Insert (polyethylene terephthalate 8.0 µm) was purchased from Millipore Corporation (Billerica, MA). The cell counting kit-8 (CCK-8) was purchased from Dojindo Laboratories (Dojindo, Japan).

### Co-culture Cell Proliferation Assay

THP-1 cells (5×10^5^/ml) were treated with different concentrations of TWEAK (0, 50, 100 and 200 ng/ml) in serum-free media for 24 h, and the conditioned media were collected and added to HO-8910PM cells in 96-well cell culture plates (1×10^5^/well), and the cells were incubated for 24 h, 48 h or 72 h. Cell proliferation was measured by the CCK-8 assay kit. Briefly, 10 µl of CCK-8 reagent was added to each well after the specified time points, and absorbance was measured at 450 nm 1.5 h later.

### Cell Matrigel Invasion Assay

Millicell Hanging Cell Culture Insert chambers were coated with 50 µl Matrigel (diluted 1∶3 in serum-free medium) and placed into 24-well plates. Human HO-8910PM cells were cultured to 70∼80% confluence before serum-starvation. Cells were then trypsinized, resuspended, and 5000 cells were placed in each insert chamber. Meanwhile, THP-1 cells (5×10^5^/ml) were treated with different concentrations of TWEAK (0, 50, 100 and 200 ng/ml) in serum-free media for 24 h, and the conditioned media were collected and added to the chambers. Media containing with 20% FBS was placed in the outer chambers, the plates were incubated for 24 h, and the cells invaded through the Matrigel membrane were fixed, stained and counted under a light microscope at 200x magnification.

### Real Time PCR

Total RNA from TWEAK-treated HO-8910PM cells was isolated using TRIzol Reagent (Invitrogen, Carlsbad, CA) and 1 µg total RNA was used as a template for cDNA synthesis using the ReverTra Ace ® Qpcr RT Kit (Toyobo, Japan). The expression of monocyte chemoattractant protein-1 (MCP-1) mRNA and the internal control GAPDH (Glyceraldehyde-3-phosphate dehydrogenase) were quantified and analyzed using the Applied Biosystems Real-Time PCR System (Carlsbad, CA, USA) with SYBR Green (Toyobo, Osaka, Japan). The primers for MCP-1 were forward: 5`-ACCACCTGGACAAGCAAACC-3` and reverse: 5`-GGGGAAAGCTAGGGGAAAAT-3`. The primers for GAPDH were forward: 5`-GAAGGTGAAGGTCGGAGTC-3` and reverse: 5`-GAAGATGGTGATGGGATTTC-3`.

### ELISA Assay

After indicated treatment/s, the cell culture supernatants was collected and analyzed using specific enzyme-linked immunosorbent assays (ELISA) kit (R&D Systems, Minneapolis, MN) for MCP-1 and TNF-α, according to the manufacturer’s instructions.

### Statistical Analysis

**Figure 1:**
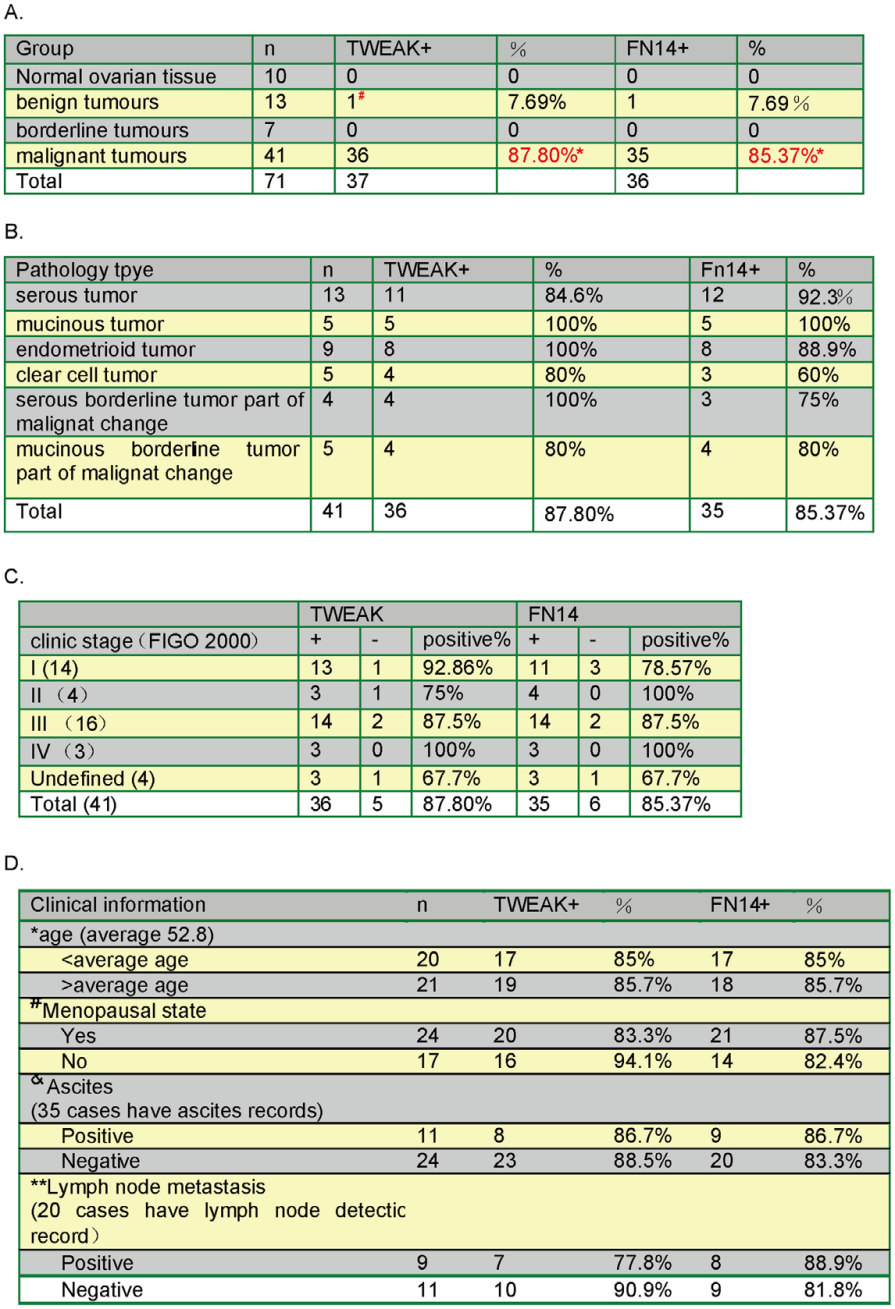
In malignant ovarian tumors, TWEAK/Fn14 expression was not corrected with patients’ clinical subtype/stages or pathological features. (A) The expression profile of TWEAK and Fn14 in different ovarian tissues (*p<0.0001, χ^2^ test, α = 0.05). (B) The expression profile of TWEAK and Fn14 in malignant ovarian tumors with different pathological type. (C) The expression profile of TWEAK and Fn14 in malignant ovarian tissues with different clinical stage (based on FIGO 2000). (D) The expression profile of TWEAK and Fn14 in malignant ovarian tissues of patents with different clinical information.

**Figure 2:**
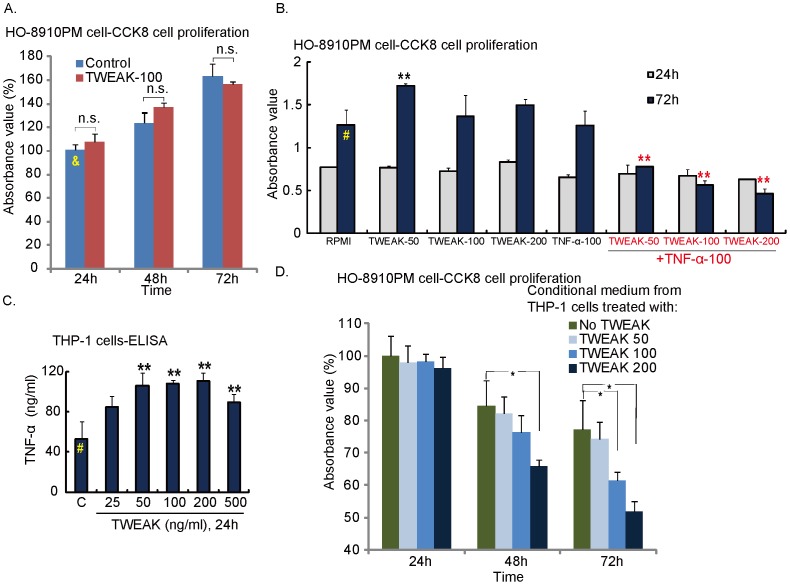
Conditioned media of TWEAK-treated THP-1 macrophages inhibits HO-8910PM cell proliferation. *(A) TWEAK itself has no effects on HO-8910PM cell proliferation.* Cultured HO-8910PM cells were either left untreated or treated with TWEAK (100 ng/ml) for 24, 48 and 72 hours, cell proliferation was measured by the CCK-8 assay. Absorbance value was expressed as percentage change of group marked as “&”. “n.s.”: non-statistical difference. *(B) TWEAK and TNF-α co-administration inhibits HO-8910PM cell proliferation.* Cultured HO-8910PM cells were either left untreated (“C”) or treated TNF-α (100 ng/ml) with or without indicated concentration of TWEAK for 24 and 72 hours, cell proliferation was measured by the CCK-8 assay. **P<0.05 vs. group “^#^”. *(C) TWEAK promotes TNF-α secretion in cultured THP-1 macrophages.* Cultured THP-1 cells were treated with indicated concentration of TWEAK for 24 h. TNF-α secretion was analyzed by ELISA. **p<0.05 vs. group “^#^”. *(D) Conditioned media from TWEAK-treated THP-1 macrophages inhibits HO-8910PM cell proliferation.* HO-8910PM cells were either cultured in RPMI or in conditional medium from TWEAK (different concentration)-treated THP-1 cells, cell proliferation was measured by the CCK-8 assay. Absorbance value was expressed as percentage change of group marked as “&”. *p<0.05.

**Figure 3:**
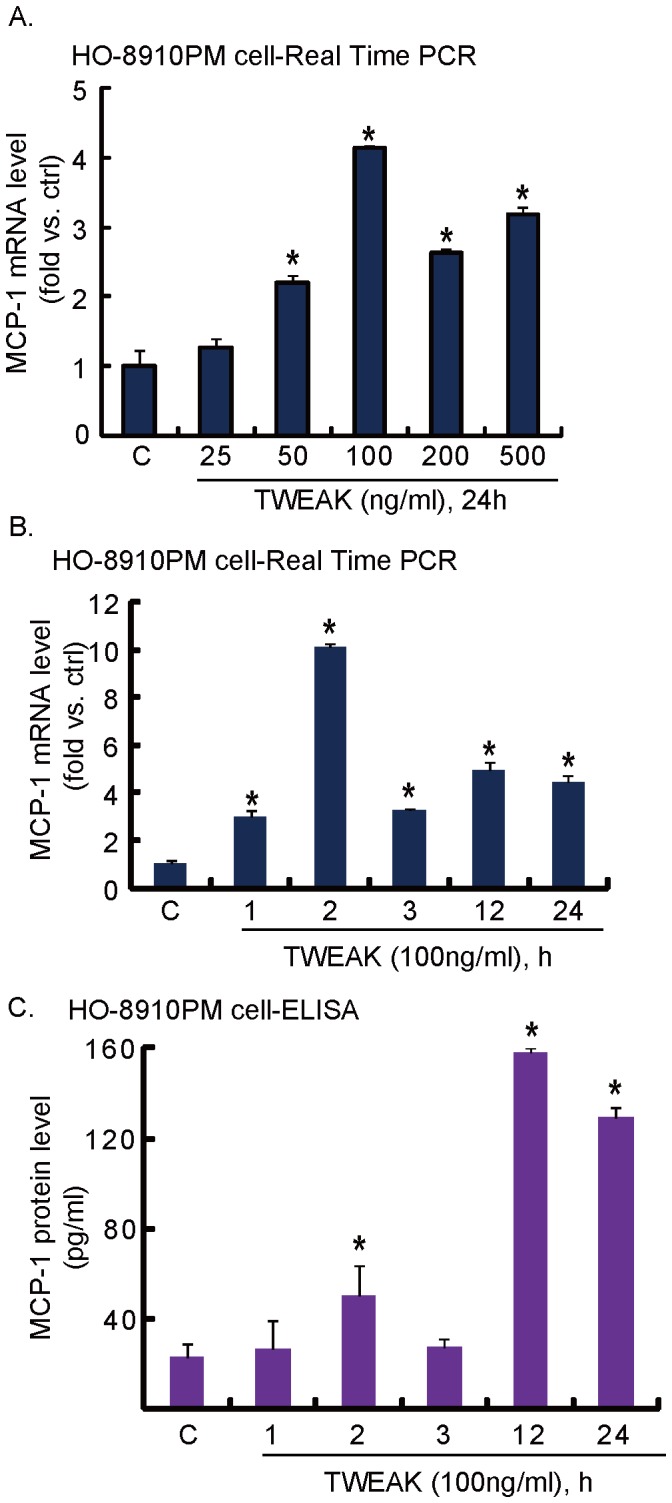
TWEAK induces MCP-1 production in cultured HO-8910PM cells. *(A–B) TWEAK increases MCP-1 mRNA levels in cultured HO-8910PM cells.* Cultured HO-8910PM cells were either left untreated (“C”) or treated with various concentration of TWEAK (25, 50, 100, 200 and 500 ng/ml) for 24 hours (A), or treated with 100 ng/ml of TWEAK and cultured for various time points (1 h, 2 h, 3 h, 12 h and 24 h) (B), MCP-1 mRNA was analyzed by real time PCR, and the value was normalized to untreated group. *p<0.05 vs. group “C”. *(C) TWEAK increases MCP-1 protein secretion in cultured HO-8910PM cells.* Cultured HO-8910PM cells were either left untreated (“C”) or treated with 100 ng/ml of TWEAK and cultured for various time points (1 h, 2 h, 3 h, 12 h and 24 h), concentration of MCP-1 (pg/ml) in culture medium was determined by ELISA assay. *p<0.05 vs. group “C”.

**Figure 4:**
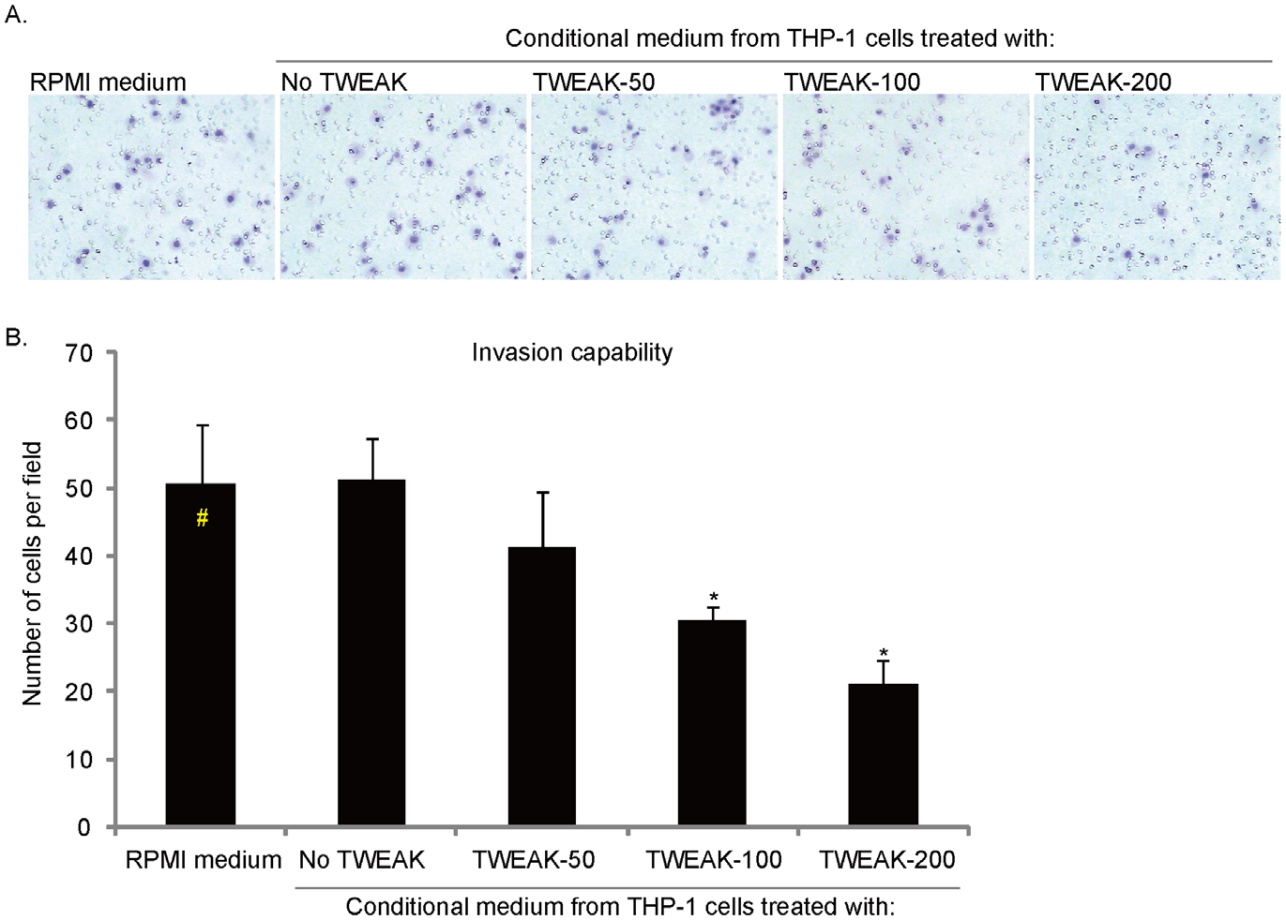
Conditioned media from TWEAK-treated THP-1 macrophages inhibits HO-8910PM cell *in-vitro* invasion. HO-8910PM cells were either cultured in RPMI (group “#”) or in conditional medium from TWEAK (different concentration)-treated THP-1 cells for 24 hours, *in-vitro* cell invasion was measured by Matrigel invasion assay, and reprehensive images was shown in (A) (1∶200), the total number of cells which migrated through the Matrigel-coated membrane in each microcopy field was recorded (B). *p<0.05 vs. group “#” (B).

For clinical samples, (Figure 1), variance was analyzed using the variance method (α = 0.05); numerical data was analyzed using the χ^2^ test (α = 0.05) with the SPSS 16 statistical analysis software package. For *in-vitro* experiments (Figures 2, 3, 4), individual culture dishes or wells were analyzed separately (no pooling of samples was used). In each experiment, a minimum of three wells/dishes was used and similar results were obtained. Each experiment was repeated a minimum of three times, the mean value of the repetitions was calculated and this value was used in the statistical analysis. Data are presented as mean ± SD. The differences were determined by one-way ANOVA in appropriate experiments followed by Newman–Keuls post hoc test. A probability value of *p*<0.05 was taken to be statistically significant.

## Results

### TWEAK is Expressed in Malignant Ovarian Tumors, but not in Normal Ovarian Tissues or in Borderline and Benign Tumors

**Figure 5:**
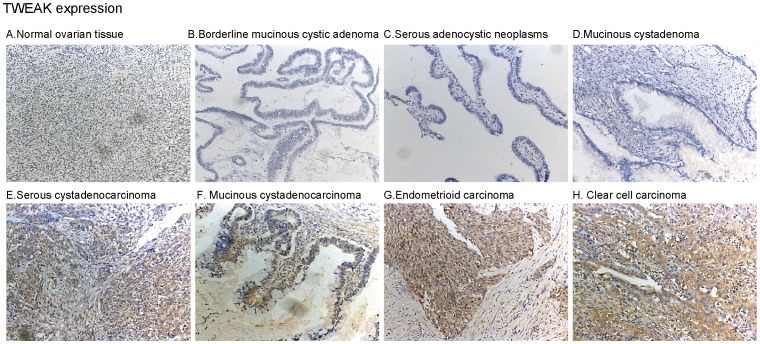
TWEAK is expressed in malignant ovarian tumors, but not in normal ovarian tissues or in borderline and benign tumors. Expression of TWEAK in different human ovarian tissues (A–H).

The ovarian tumor tissues in this study were obtained from 41 patients with malignant epithelial ovarian tumors (13 serous carcinomas, 5 mucinous carcinomas, 9 endometrioid carcinomas, 5 clear cell carcinomas and 9 borderline tumors with malignant changes), 7 patients with borderline epithelial ovarian tumors and 13 patients with benign epithelial ovarian tumors (See Figure 1A). Normal ovarian tissues (n = 10) were obtained from patients undergoing hystero-salpingo-oophorectomy. We performed IHC to detect TWEAK expression in different ovarian tissues as described, and observed that TWEAK was expressed at high levels only in malignant tumors (Figure 5E–H). We didn’t see significant TWEAK expression in normal ovarian tissues, or in borderline/benign tumors (Figure 5A–D). These results suggest that TWEAK is uniquely expressed only in malignant ovarian tumor tissues.

### TWEAK Receptor Fn14 is Expressed in Malignant Ovarian Tumors, but not in Normal Ovarian Tissues or in Borderline and Benign Tumors

**Figure 6:**
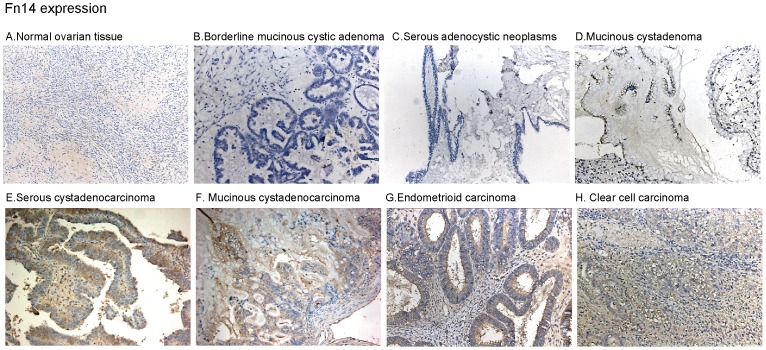
Fn14 is expressed in malignant ovarian tumors, but not in normal ovarian tissues or in borderline and benign tumors. Expression of Fn14 in different human ovarian tissues (A–H).

Using the same IHC method, we then examined the expression of Fn14, the TWEAK receptor, in the ovarian tissues described above. Again, as shown in Figure 6E–H, a significant high level of Fn14 was observed only in malignant ovarian tumors, while in normal ovarian tissues, borderline/benign epithelial ovarian tumors, its expression level was very low (Figure 6A–D). These results suggest that TWEAK receptor Fn14 is also uniquely expressed in malignant ovarian tumors.

### In Malignant Ovarian Tumors, TWEAK/Fn14 Expression was not Corrected with Patients’ Clinical Subtype/Stages or Pathological Features

We then summarized the expression profiles of TWEAK and Fn14 in different types of ovarian tissues. High level of TWEAK expression was detected in the majority of malignant tumors (36 out of 41, 87.80%), where almost no TWEAK expression was observed in normal ovarian tissues or in borderline and benign tumors (1 out of 30, 3.33%) (*p*<0.05). Note only 1 benign tumor was TWEAK positive (Figure 1A ^#^). Similarly, 35 out of 41 (85.37%) malignant ovarian tumors were Fn14 positive, compared to 1 out of 30 (3.33%) in normal ovarian tissues plus borderline/benign tumors (Figure 1A) (*p*<0.05). However, we didn’t see any significant correlations between the expression of TWEAK/Fn14 in the malignant ovarian tumor with the clinical subtype (Figure 1B) or stage (Figure 1C) of the patients (*p*>0.05). Further, no significant correlation between the expression of TWEAK/Fn14 in malignant tumor tissues with clinic–/pathological- features (age, menopausal state, ascites and lymph node metastasis) of the patients was observed (Figure 1D) (*p*>0.05). These data together suggest that although TWEAK/Fn14 is expressed in the majority of malignant tumors, their expressions are not correlated with patients’ clinical subtype/stages and pathological features.

### Conditioned Media of TWEAK-treated THP-1 Macrophages Inhibits HO-8910PM Cell Proliferation

Cell proliferation results in Figure 2A demonstrated that the adding TWEAK (100 ng/ml) directly to the culture media had no significant inhibitory effect on the proliferation of HO-8910PM cells. This result was not surprising as TWEAK was basically a weak inducer of cell death. Studies have reported that TWEAK-induced cell death required very long incubation periods, and could often only be detected when target cells were sensitized with cycloheximide pre-stimulation or other cytokines co-incubation [27]. For example, a recent study showed that co-administration of TNF-α and TWEAK induced cell death in primary and transformed keratinocytes, while neither TNF-α or TWEAK had a significant effect [27]. Further, it has been shown that TWEAK diminished TNF-α–induced pro-survival NF-κB pathway activation [28], while NF-κB inhibition was known to sensitize TNF-α–induced apoptosis [29]. We also found that TWEAK and TNF-α simultaneous exposure induced the maximal HO-8910PM growth inhibition (Figure 2B), while either TWEAK or TNF-α had almost no such effects (Figure 2A–B). ELISA results in THP-1 macrophage cell line showed that TWEAK promoted TNF-α production (Figure 2C). Since the condition medium from TWEAK stimulated THP-1 cells should contain both TWEAK and TNF-α, we proposed that this condition medium might be able to inhibit ovarian cancer cell proliferation. As a matter of fact, we observed a significant cell growth inhibition when HO-8910PM cells were cultured in the conditioned media from TWEAK-treated THP-1 cells (Figure 2D). The effect appeared to be dose-dependent, as the conditioned media from 200 ng/ml TWEAK-treated THP-1 cells showed the most significant effect (Figure 2D).

### TWEAK Induces MCP-1 Production in Cultured HO-8910PM Cells

The proposal for the current study is that TWEAK activates tumor cells to recruit and activate macrophages, which then inhibit tumor cell growth and invasion. MCP-1, a member of the C-C chemokine family, is a potent chemotactic factor for monocytes. MCP-1 can be induced by stimulations including oxidative stress, cytokines or growth factors in a number of cell types (including tumor cells). MCP-1 is the main chemokine to recruit monocytes to the site of active inflammation [30], [31]. We next examined the effect of TWEAK on the expression and production of MCP-1 in HO-8910PM cells (Figure 3). The real-time PCR was used to test MCP-1 mRNA level in HO-8910PM cells treated with TWEAK, and results showed that TWEAK significantly increased MCP-1 mRNA level in HO-8910PM cells (Figure 3A and B), and 100 ng/ml of TWEAK demonstrated most significant effects (Figure 3A). TWEAK mRNA level started to increase at 1 h after TWEAK treatment and lasted for at least 24 hours (Figure 3B). The ELISA results showed that TWEAK also promoted MCP-1 production in cultured HO-8910PM cells, as MCP-1 protein level increased significantly after 100 ng/ml of TWEAK stimulation (Figure 3C). These results suggested that TWEAK upregulated MCP-1 mRNA and induced its secretion in cultured HO-8910PM cells, which might recruit macrophages.

### Conditioned Media from TWEAK-treated THP-1 Macrophages Inhibits HO-8910PM Cell in-vitro Invasion

The cellular Matrigel invasion assay results demonstrated that the supernatant of TWEAK-treated THP-1 cells inhibited the invasive ability of HO-9810PM cells *in-vitro* (Figure 4). The conditioned media from TWEAK-treated THP-1 cells reduced the invasion ability of HO-8910PM cells (Figure 4A and B). The effect appeared to be dose-dependent, and media from with 200 ng/ml of TWEAK-treated THP-1 cells showed most significant inhibitory effects on HO-8910PM cell invasion (Figure 4B).

## Discussion and Conclusion

Normally, TWEAK is expressed on the activated monocytes and T cells. However, its expression increases significantly in tumor tissues during cancer progression [6], [32], which is associated with infiltration of inflammatory cells, and activation of resident innate immune cells [17]. TWEAK and its receptor Fn14 mRNA and/or protein expression were seen in multiple cancers tissues including human glioma [13], lung carcinoma [1], and HCC [6], as well as in various cultured cancer cell lines [17], [32]. In consistent with these studies, we found that high level of TWEAK expression was detected in the majority of malignant tumors (36 out of 41, 87.80%). Similarly, 35 out of 41 (85.37%) malignant ovarian tumors were Fn14 positive. Although in these malignant ovarian tumors, TWEAK/Fn14 expression was not corrected with patients’ clinical subtype/stages or pathological features. The unique expression of TWEAK and Fn in malignant ovarian tumors suggest that they can be potentially used as a malignant tumor marker.

The expression profile of TWEAK/Fn in human ovarian cancer tissues implies that the TWEAK present in the tumors may regulate tumor cell progression. As a matter of fact, soluble TWEAK could act on tumor cells via an autocrine or paracrine regulatory mechanisms, while membrane-anchored TWEAK acting via a juxtacrine mechanism [17].The role of TWEAK on tumor cells is inconclusive, as some studies show that TWEAK alone is able to promote cell death (cell apoptosis and/or necrosis) in human HT29 colon adenocarcinoma cells [33], human HSC3 oral squamous cell carcinoma cells [3], [8] and rhabdomyosarcoma cells [34], while others show that TWEAK promotes cancer cell proliferation [6]. It is now well-accepted that TWEAK was basically a weak inducer of cell death and apoptosis, here we showed that the directly adding of TWEAK (100 ng/ml) to the culture media had no significant inhibitory effect on the proliferation of HO-8910PM cells for at least 72 hours, however, TWEAK and TNF-α co-administration induced the maximal HO-8910PM cell growth inhibition. Meanwhile, medium from TWEAK-treated THP-1 cells, which contained both TWEAK and TNF-α, also significantly inhibited HO-8910PM cell proliferation. These results indicate that TWEAK/Fn14 might be a tumor suppressor in ovarian tumors, and TWEAK may activate tumor associated macrophages to inhibit cancer cell progression. In support of this, we observed a significant MCP-1 expression in TWEAK-treated HO-8910PM cells, which is a major chemokine to recruit macrophages. The cooperative effect of tumor derived-TWEAK and macrophage (activated by TWEAK) derived-cytokines may inhibit the proliferation and invasion of ovarian tumor cells, and may even induce tumor cell death.

The human immune system plays a crucial role in the process of tumor rejection. Most chemotherapy regimens attempt to enhance the antigenicity of tumor cells and make tumor cells more detectable by the immune system. Maecker et al. suggested that TWEAK was an important regulator of the innate immune system [35]. Expression of TWEAK and Fn14 can be upregulated by various stimuli in the cells involved in innate immunity. Previous studies showed that TWEAK could regulate the activation of the cells involved in innate immunity, including NK-cells, dendritic cells and macrophages [35], these cells infiltrate into tumor microenvironment and affect tumor growth and invasion. Kaduka et al. observed that TWEAK mediates an anti-tumor effect similar to tumor-infiltrating macrophages [21]. These observations suggested that TWEAK could directly modulate the function of the innate immune system, and thereby indirectly influenced tumor progression [35]. We suggested that TWEAK could potentially be used as a novel therapeutic intervention to regulate the innate immune system of patients with advanced ovarian cancer, by activating the innate immune system, thereby improving the prognosis of patients with ovarian cancer.
