# Correction: Functional Expression of TWEAK and the Receptor Fn14 in Human Malignant Ovarian Tumors: Possible Implication for Ovarian Tumor Intervention

**DOI:** 10.1371/annotation/aa15cad6-0a7a-4b4e-ac1b-b24af35b723c

**Published:** 2013-03-12

**Authors:** Liying Gu, Lan Dai, Cong Cao, Jing Zhu, Chuanwei Ding, Hai-bo Xu, Lihua Qiu, Wen Di

There was an error in the first affiliation. The correct affiliation is:

"Department of Gynecology and Obstetrics, Ren Ji Hospital, School of medicine, Shanghai Jiao Tong University, Shanghai, P.R. China" 

